# The Impact of Corticosteroid Administration at Different Time Points on Mucosal Wound Healing in Rats: An Experimental Pilot In Vivo Study

**DOI:** 10.3390/biology11091309

**Published:** 2022-09-02

**Authors:** Evgeny Weinberg, Nirit Tagger-Green, Michal Lusthaus, Marilena Vered, Eitan Mijiritsky, Liat Chaushu, Roni Kolerman

**Affiliations:** 1 Department of Periodontology and Oral Implantology, The Goldschleger School of Dental Medicine, Sackler Faculty of Medicine, Tel Aviv University, Tel Aviv 69978, Israel; 2 Department of Oral Pathology, Oral Medicine and Maxillofacial Imaging, The Goldschleger School of Dental Medicine, Sackler Faculty of Medicine, Tel Aviv University, Tel Aviv 69978, Israel; 3 Head and Neck Surgery and Maxillofacial Surgery Department, ENT Array, Tel-Aviv Sourasky Medical Center, Sackler School of Medicine, Tel-Aviv University, Tel Aviv 64239, Israel; 4 Goldschleger School of Dental Medicine, Sackler School of Medicine, Tel-Aviv University, Tel Aviv 69978, Israel

**Keywords:** stem cells, corticosteroids, palatal wound healing

## Abstract

**Simple Summary:**

The objective of this pilot study was to evaluate the impact of corticosteroid (CS) administration at different time points on palatal wound healing in rats. Thirty-six young male rats were divided into three groups. The test groups were treated by CS in the early (1–4 days) and late (5–9 days) stages after palatal wounding, while the control group was left for spontaneous healing. Our findings do not support the positive impact of CS administration on palatal wound healing. While microscopically, we found no difference between the CS and control groups, CS exposure was associated with a macroscopically larger final wound area, reflecting a possible harmful effect of CS.

**Abstract:**

**Background:** Conflicting results were found regarding the effect of corticosteroid (CS) administration upon wound healing. The objective of this pilot study was to evaluate the impact of CS administration at different time points on palatal wound healing in rats. **Methods:** A 4.2 mm diameter punch created a secondary healing excisional palatal defect in thirty-six (36) Wistar-derived, two-month-old male rats weighing 250–270 g. We evaluated the effect of CS by comparing wound healing between three equal groups: 12 rats who were not exposed to CS and two additional groups in which 1 mg/kg dexamethasone (1 mg/kg) was administered daily, early (1–4 days) and late (5–9 days) after injury. The dynamics of the healing process were evaluated weekly in 4 sacrificed rats from each group for three weeks. The wound area was assessed both macroscopically and microscopically; the inflammation score was assessed microscopically. **Results:** The initial wound area in all the rats was 13.85 mm^2^. At the end of the study, it decreased to 4.11 ± 0.88 mm^2^, 7.32 ± 2.11 mm^2^, and 8.87 ± 3.01 mm^2^ in control, early, and late CS administration groups, respectively (*p* = 0.075). Inflammation scores showed a tendency to decrease in the third week in all groups, with no statistical differences. **Conclusions:** Our findings do not support the positive impact of CS administration on palatal wound healing. While microscopically, we found no difference between the CS and control groups, CS exposure was associated with a macroscopically larger final wound area, reflecting a possible harmful effect of CS.

## 1. Introduction

The hard palate is a source for free gingival graft (FGG) or connective tissue grafts (CTG) harvesting, used in mucogingival surgery to treat gingival recession. The harvesting site usually heals within 14–28 days [1]. The FGG harvesting technique is associated with common side effects, including distress, bleeding, aching, swelling, difficulty chewing, eating, speaking, unpleasant smell, infection, and loss of sensation [2].

The palatal excisional wound in the rat is a reproducible model for evaluating clinical and histological outcomes. Many studies used this model to investigate factors that might enhance intra-oral wound healing [3,4,5,6,7,8,9], such as topical use of biomaterials [5,6,7,8], antimicrobials [9], and growth factors [10]. Other factors that have been found to impact wound healing, probably by modulating the inflammatory process, include age and gender [4,11,12,13], systemic treatment with corticosteroids [14,15,16,17,18,19,20,21] or retinoids [15], and pathological conditions such as Xerostomia [22,23,24] and diabetes mellitus [25].

The healing of excisional wounds with a significant palatal soft-tissue defect largely depends on individual environmental factors, unlike minor injuries characterized by a better healing potential [22,23,24]. Compared to dermal tissue, mucosal tissue heals much quicker with less inflammation and scarring [26,27]. This suggests that the level of inflammation needed for optimal healing is lower in mucosal tissue. The previous statement was supported by an earlier study demonstrating faster clinical wound healing in the old male rats than the young rats due to diminished and delayed inflammatory response in the aged rats [4].

The effects of CS on wound healing have greatly interested surgeons, internists, and dermatologists. Within the past century, pivotal discoveries in wound healing mechanisms have enhanced our understanding of the molecular interactions between corticosteroids and cutaneous wounds [14]. Corticosteroids have been shown to affect all major steps of the wound healing process, namely the inflammatory, proliferative (tissue formation), and tissue remodeling phases [15,16]. These three stages overlap, as signaling cascades started in the first phase influence cell growth and differentiation in later stages [17].

During the Inflammatory phase, dexamethasone treatment decreases cytokine expression, including TGF-b1, platelet-derived growth factor, tumor necrosis factor, and interleukin-1a in wounded tissue [18]. It may reduce other inflammatory cells’ chemotactic and mitogenic stimulus [18]. Dexamethasone also downregulates endothelial cell expression of intercellular adhesion molecule 1 (ICAM-1) in culture resulting in attenuated granulocyte adhesion and migration [19]. Consistent with this finding, high-dose CS reduces the extent of macrophage infiltration into the wound [20].

During the proliferative phase, CS administration reduces TGF- β 1 levels and mesenchymal cell expression of the keratinocyte growth factor (KGF), which attenuates fibroblast proliferation [14] and impairs wound re-epithelialization [21].

Conflicting results exist regarding CS administration and wound healing [28,29,30]. Regarding the timing of administration, animal studies have shown that corticosteroids given at least three days after wounding do not affect wound healing [28]. Controversially systemic CS administration improved tendon healing when given after the early inflammatory phase [29]. Therefore, local treatment might have different effects from systemic glucocorticoid treatment, which act on the immune system, including the bone marrow. There seems to be scarce data on systemic glucocorticoid treatment and palatal wound healing.

The present comparative study aims to assess the timing of early or delayed corticosteroid administration on the palatal wound healing of young male rats.

## 2. Materials and Methods

### 2.1. Animals and Preparation of Experimental Model

The Ethics and Institutional Animal Care and Use Committee of Tel Aviv university approved the study (no-034-b17858-22).

The study design followed the Animal Research Reporting In Vivo Experiments (ARRIVE) guidelines [31]. The study group consisted of 36 Wistar-derived, two-month-old male rats weighing 250–270 g. All surgical procedures were performed by the same experienced operator (EW). At time 0 (W0), a circular excisional wound 4.2 mm in diameter was made in the center of the palatal mucosa using a tissue punch producing a wound area of 13.85 mm (initial wound area). After removing the soft tissue, a circular area of denuded bone (without periosteum) was left for secondary healing [32]. The protocol of surgery, aftercare, and sacrificing techniques, as well as macroscopic and microscopic assessments, are described in detail in our previous publication [4]. Animals were randomly sacrificed at one week (W1), two weeks (W2), and three weeks (W3) post-operatively. The maxillae were detached and shifted for fixation into 10% buffered formalin for one day.

### 2.2. Steroid Administration

The rats were divided according to the day of sacrifice into three groups of 12 each.

Study group A: Early Dexamethasone (Early Dex) administration, days 1–4 (daily) after surgery.

Study group B: Late Dexamethasone (Late Dex) administration, days 5–9 (daily) after surgery.

Control group: No steroid administration.

Calculation of Dexamethasone dose:

- Five bottles of 1 mL each with a concentration of 10 mg/mL were used.
- 0.5 mL was aspirated from each bottle and diluted with 4.5 mL saline.
- Of the solution, 1 mg/kg was injected subcutaneously into the abdominal wall.

### 2.3. Macroscopic Evaluation

The palate specimens were calibrated using a 15 mm long University of North Carolina color-coded periodontal probe with millimeter markings (UNC-Hu Friedy Manufacturing Inc., Chicago, IL, USA). The specimens were photographed in a standardized manner using a Cannon EOS 550D camera (Canon Inc., Tokyo, Japan) immediately after tissue harvesting (W0) at one week (W1) and immediately after sacrifice before maxillary harvesting for histological examination, W2 and W3 [4].

ImageJ software (National Institute of Mental Health, Bethesda, Maryland, USA) was used to analyze the wound-marked boundaries using a computer magnified digital photograph (as described elsewhere), and the boundaries of the wound were determined on the magnified image [4].

**Figure 1:**
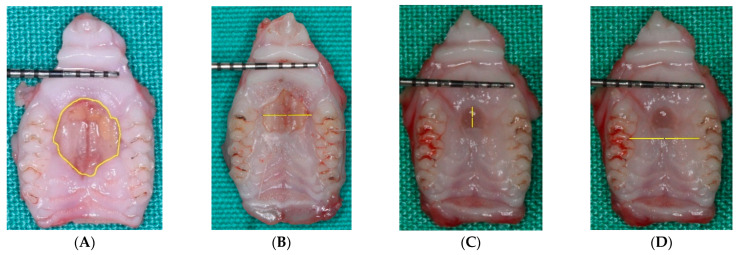
Representative photographs of the wounds using a 1 mm periodontal probe as scale measurement. (**A**) Total area in mm^2^ of the wound at one week. (**B**) Maximum L-L length in mm of the wound at two weeks. (**C**) Maximum A-P length in mm of the wound at three weeks. (**D**) Palatal width in mm (Arch distance) at three weeks.

The following measurements were taken at each time point (W1, W2, W3):

Total area, maximum L-L, and the A-P dimensions of the wound (Figure 1).

Palatal width (arch distance) was measured as the intermaxillary distance between the first and second molar contact points (Figure 1).

### 2.4. Microscopic Examination

After fixation and decalcification [4], the samples (*n* = 24) were cut in the frontal plane through the point of the maximum lateral-lateral distance of the wound and embedded in paraffin. Three micron-thick sections were prepared and stained with hematoxylin and eosin (H&E). Each of the stained slides was photographed at ×20 and ×40 magnification using a light microscope (Olympus BH-2, Tokyo, Japan) equipped with a digital camera (with the original files saved as JPEG files) [4].

### 2.5. Histomorphometry for Assessment of the Intensity of Inflammation

**Figure 2:**
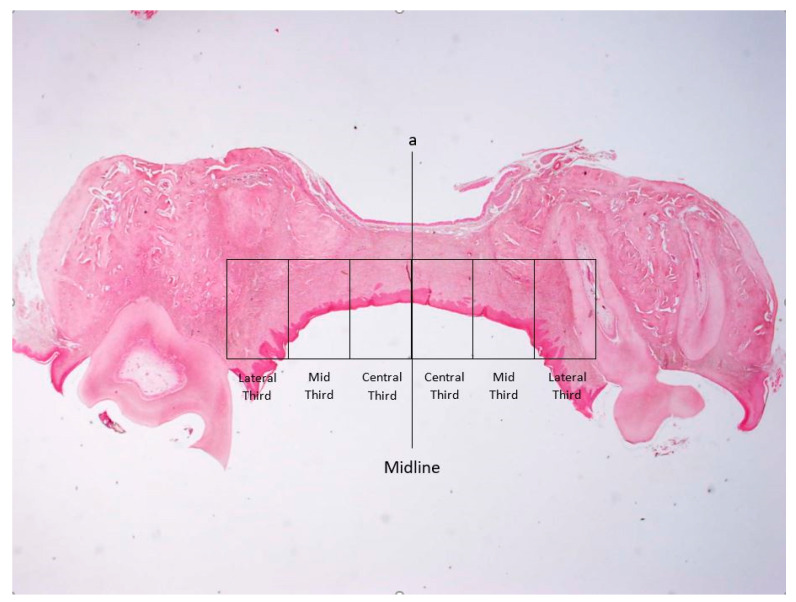
Illustration of the method used for establishing the palatal central, mid, and lateral thirds for the semi-quantitative assessment of the inflammation; a—the vertical line drawn at the midline of the palate (Hematoxylin and eosin stain, ×20 original magnification).

The method used to assess the intensity of inflammation was previously described by our group [4]. Briefly, a vertical line was drawn at the midline of the palate. Two perpendicular horizontal lines were drawn parallel to the surface of the wound: one was between the palatal aspects of the alveolar ridges, and the second was beneath the nasal mucosa. These lines were first divided into equal halves, and then each half (right and left to midline) was further divided into three equal parts, thus creating equal central, mid, and lateral rectangles, which were termed “thirds” (Figure 2) [32]. A semi-quantitative evaluation of the intensity of the inflammatory response was performed so that each third was assessed using a 0 to 4 score system as follows: 0 = no inflammatory cells; 1 = a few inflammatory cells; 2 = similar to “1” with the addition of small foci consisting of 10 cells all over the examined third; 3 = identical to “2” with foci comprising >10 cells all over the examined third [33]. Results were presented as the mean inflammation score for each third at each time point.

### 2.6. Statistical Analysis

Figures were evaluated using SPSS version 24 (SPSS Inc., Chicago, IL, USA). For statistical analysis of the macroscopic wound measurements at different time points (Weeks 1, 2, and 3) within three groups (early CS, late CS, and control), two-way analyses of variance (ANOVA) were used. A significant interaction may indicate that the wound’s size change over time depending on the administration of CS. ANOVA with repeated measures of within-subjects and between-subjects effects was used to analyze statistical significance for mean inflammation scores.

## 3. Results

### 3.1. Macroscopic Evaluation

Time 0: The initially created wound area was 13.85 mm^2^ (initial wound area), as described previously. The macroscopic healing at W1, as assessed by calculating the total wound area, showed minimal progression relative to the surgical phase (W0).

**Figure 3:**
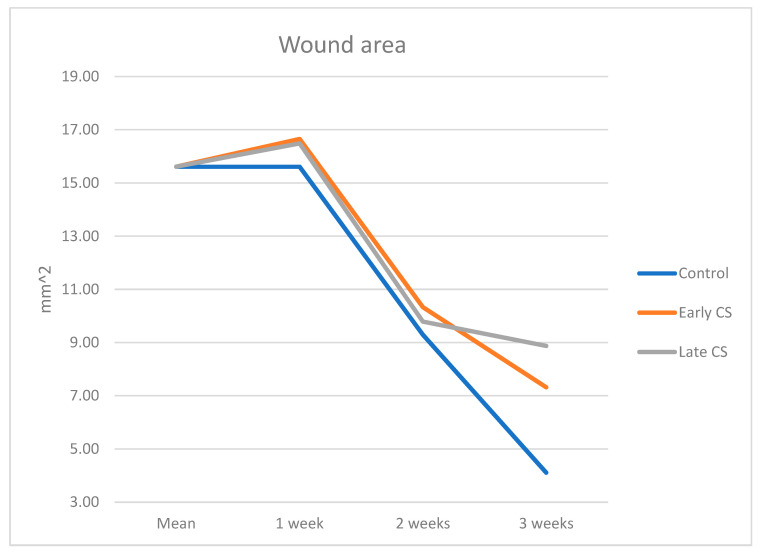
Macroscopic wound area in the test and control groups related to time.

One week: Total wound area (Table 1, Figure 3) was similar between the Early CS group (16.66 ± 3.43 mm^2^) and the Late CS group (16.49 ± 2.16 mm^2^), and the control group (15.61 ± 2.54 mm^2^) (*p* = 0.851).

Two weeks: Total wound area in the Early CS group was 10.33 ± 4.80 mm^2^, Late CS group 9.79 ± 2.45 mm^2^, and 9.29 ± 1.54 mm^2^ in the control group (*p* = 0.903).

Three weeks: Total wound area in the Early CS group was 7.32 ± 2.11 mm^2^, Late CS group 8.87 ± 3.01 mm^2^, and 4.11 ± 0.88 mm^2^ in the control group (*p* = 0.075) with no statistical significance between the groups.

During the three weeks, the total area decreased continuously among the two tests and control arms (Table 1, *p* = 0.17). The three weeks presented better healing (although incomplete) in the control group compared to both test groups, but the differences did not reach statistical significance. (Table 1 and Figure 3).

In the L-L and A-P dimensions, a significant reduction was found between 1, 2, and 3 weeks. (Table 1). No statistical differences were found between the three groups regarding the L-L, A-P, and palatal width parameters.

### 3.2. Microscopic Evaluation

Results in the test and control groups (Table 2) showed a significant decrease (*p* < 0.000) from W2 to W3 in all thirds. No statistical differences existed between test groups and control in the different zones nor as a function of time.

**Figure 4:**
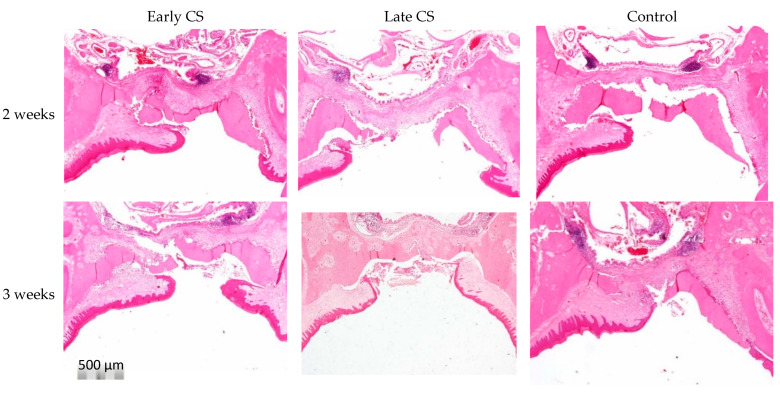
Representative photomicrographs of the wound region at two and three weeks. At two weeks, there was more inflammation compared to the third week. Mean scores are detailed in Table 2. Full epithelization was not completed by the third week (Hematoxylin and eosin stain, original magnification ×40).

**Figure 5:**
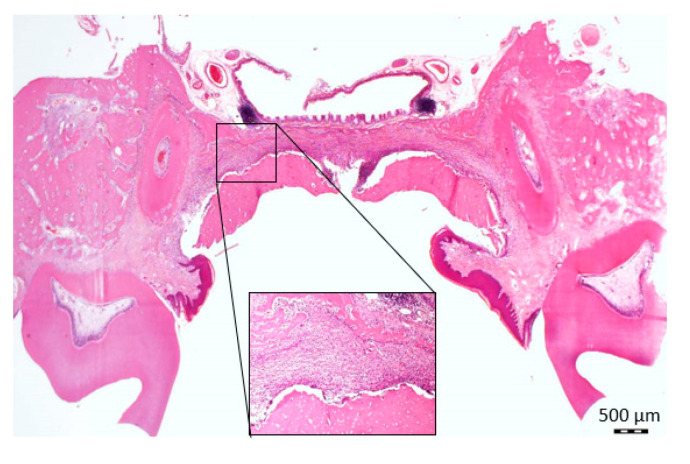
Microscopic photomicrograph of a control rat at two weeks presents incomplete epithelialization and granulation tissue (see inset) (Hematoxylin and eosin stain, original magnification ×20).

At two weeks, the wound showed granulation tissue that was composed mainly of monocytes, granulocytes, fibroblasts, blood vessels, and the extracellular matrix (Figure 4 and Figure 5). At three weeks, the inflammatory infiltrates decreased in all thirds compared to the second week (Figure 4). Most rats did not complete full epithelization by the third week (Figure 4).

## 4. Discussion

In the present study, the healing pattern of the palatal wound in young male rats was not affected by daily administration of corticosteroids either in the early (1–4 days after surgery) or at the late stage (5–9 days after surgery) after injury compared to the control group (no medication). At the final measurement at three weeks, better healing (although incomplete) in the control group compared to both test groups was observed, although the differences did not reach a statistical significance; thus, CS administration seemed to damage the healing compared to the control group. It has commonly been reported that people heal more slowly with increasing age [12]. However, a chief criticism of such findings is that studies have not adequately controlled for confounding factors that are more common in aged persons, such as medication use and morbidities [12].

The present study was performed using young male rats. The amplified inflammatory response in young rats may be responsible for our finding of retarded healing response. Our results of a decrease from an initial wound area of 13.85 mm^2^ to a final wound area of 4.11 ± 0.88 mm^2^ in the control rats are in accordance with the findings in our previous study showing three weeks last wound healing area of 4.89 ± 1.60 mm^2^ in 2-month young rats [4]. In both studies, the defect was progressively occupied with soft tissue during the proliferative and remodeling phase in the test and control groups. However, whole wound closure was not achieved in any of the groups in the present study, similar to the findings in young rats but contrary to the complete healing observed in the old rats at three weeks post-surgery in our previous publication [4]. In our study, we used only male rats, as gender has been found to play a role in the healing process. To date, wound healing studies have mainly examined dermal wounds and reported female advantages in healing rates [34,35,36,37]. Conversely, when observing oral mucosal injuries, studies have found a male advantage in healing rates [12]. In addition, after oral surgical procedures, mucosal wound healing was associated with more significant complications and longer recovery in women [38,39,40,41]. Thus, gender advantages in wound healing seem to be tissue specific. Sex hormones, specifically estrogens, and progesterone, play a role in mucosal inflammation, as confirmed in gingivitis [42] and periodontal disease [43]. Notably, the sexual dimorphism observed in dermal healing rates has been linked to the modulating effects of sex hormones on healing processes, specifically on inflammation [34,35,37]. Overall, androgens generally lengthen, whereas estrogens shorten healing times in the skin [37].

Experimental wound healing has been vastly studied [44]. It is a dynamic, interactive process involving soluble mediators, blood cells, extracellular matrix, and parenchymal cells. Oral mucosal and skin wound healing follows a similar pattern of inflammation, proliferative (tissue formation), and tissue remodeling phases [44]. The wound is cleared of debris and bacteria by neutrophils and macrophages during inflammation. In addition, myeloid cells are recruited to the wounded tissue, of which the monocytes differentiate into macrophages [45]. Next, neo-epithelialization and granulation tissue formation occur in the tissue formation phase. Cells from the surrounding tissue, including local stem cells, are activated and invade the wound bed [46]. Upon tissue damage, circulating bone marrow-derived cells (BMDCs) are recruited to the wound and can differentiate into tissue-specific cells [47]. Finally, in the remodeling phase, part of the fibroblasts differentiates into myofibroblasts, which can also originate from BMDCs [48]. Myofibroblasts possess contractile properties and are mainly responsible for wound contraction. Those cells also deposit large amounts of collagen and then go into apoptosis, ultimately leaving behind an acellular scar [49,50].

The modulation of inflammatory reaction using CS during the inflammatory phase that prevails during the first three days was analyzed in young rats like in the present study [51]. It was shown that an acute administration of CS causes at least a 50% decrease in the total circulating WBCs at the surgical wound site on postoperative day 1 [51]. Periodontal disease is caused by subgingival bacterial species that adversely affect the host immune system that creates and maintains unmitigated inflammation in gingival and periodontal tissues. Currently, there is a shifting paradigm in the pathogenesis of periodontitis as more studies have confirmed that periodontal disease results from exaggerated inflammatory reactions [52]. As so, encouraging results were found following the administration of host modulation therapy (HMT) in periodontal disease [53].

The present study did not find differences between the test and the control groups. Our findings may be explained by the timing and route of administration of CS or by the dosage. The preponderance of human literature found that high-dose CS administration for10 days has no clinically important effect on wound healing. In patients taking chronic corticosteroids for at least 30 days before surgery, their rates of wound complications may be increased 2 to 5 times compared with those not taking CS [54]. Literature has shown that prolonged administration of corticosteroids, both locally and systemically, inhibits inflammatory processes, which may predispose patients to delayed healing and decreased tensile strength in wounds. During the remodeling phase, the wound undergoes contraction and alters its collagen expression pattern [44]. Animal studies suggest that corticosteroids impair wound healing affects collagen turnover and disrupts the dermal-epidermal junctional interactions by decreasing the tensile strength of cutaneous wounds by reducing collagen accumulation [54].

A previous rat study [28] suggested that CS does not affect wound tensile strength if administered three or more days after wounding. This can explain the lack of benefits regarding the healing in the late CS group (administration on days 5–9).

Regarding the timing of CS administration, a previous review evaluated the outcome of acute CS administration on wound tensile strength in animals (primarily rats) [54]. In all the reviewed studies, animals were given CS within one week before surgery and then daily after surgery until the time of outcome measure determination [54]. In each case, the outcome measure was determined within a week after wounding. The results of the previously mentioned review showed a decrease of 0–65% in tensile strength [54]. As the dose and chronicity are defined concerning hypothalamic–pituitary–adrenal axis suppression, administration lasting less than 5 days could be considered ‘‘acute,’’ and doses greater than 10 mg/kg/day of cortisol for more than one week might be regarded as ‘‘chronic” [55]. These figures estimate the endogenous levels of CS produced under physiologic conditions. Any dose lower than these values is unlikely to have pharmacologic effects. The doses administered in the studies mentioned above (only data related to rats was used) ranged between 4–100 mg/kg/day of cortisol [54]. In the present study, we used one mg/kg/day of dexamethasone, equivalent to 26 mg of cortisol. An interesting study found a dose-dependent effect of GC on wound healing, showing that administration of 100 mg/kg/day of cortisol was associated with a significantly more impaired wound healing as reflected by the reduction of tensile strength compared to 4 mg/kg/day [56]. Like the observations in humans, we noticed that the periphery of the mucoperiosteal palatal wounds in rats was filled earlier than the center of the injury.

Limitations. Being a pilot project, this study has inherent limitations; mainly, the number of animals enrolled is limited. Unfortunately, a direct implication of the current study results to humans is not feasible due to the variance between the commonly used site of connective tissue graft harvesting, between the palatal aspect of the tooth and the palatal midline in humans to the mid-central palate in the rat model. The difficulty in extrapolating our present study results to real-life practice also relies on the variation in the thickness of the graft and the vascularization [57]. Regarding the timing, in previous animal studies, the CS was administered preoperatively and then continued during the first week [54]. More clinical studies are needed to evaluate the differential effects of corticosteroids on wound healing when given before versus after surgery. As for today, there is no data to justify the application of our protocol in humans.

## 5. Conclusions

Our results highlight the dynamic process of wound healing and do not support a positive impact of CS administration on palatal wound repair. Microscopically, a decrease in the inflammatory infiltrate was observed in both test and control groups from the second to the third week. Nevertheless, full epithelialization was rarely observed, even in the third week. This could be attributed to the younger age and, consequently, the lower weight of the animals and/or to a larger initial wound area compared with other studies. CS exposure was associated with a macroscopically larger final wound area, reflecting a possible harmful effect of CS. A thorough investigation of different doses, routes of administration, and CS timing is needed to establish the optimal CS regimen for healing improvement.

## Figures and Tables

**Table 1:** Macroscopic wound area measurements (mean) as a factor of time in the distance (mm) and area (mm^2^) indicators in the early corticosteroids group (test 1) vs. late corticosteroids group (test 2) and control.

	A-P (mm)	L-L (mm)	Area (mm^2^)
Group A—Early CS			
Week 1	4.98 ± 0.71	4.63 ± 0.22	16.66 ± 3.43
Week 2	3.71 ± 1.08	3.76 ± 1.05	10.33 ± 4.80
Week 3	2.23 ± 0.55	3.21 ± 0.44	7.32 ± 2.11
Group B—Late CS			
Week 1	5.04 ± 0.70	4.41 ± 0.60	16.49 ± 2.16
Week 2	3.32 ± 0.66	3.99 ± 0.54	9.79 ± 2.45
Week 3	3.64 ± 0.97	3.60 ± 1.37	8.87 ± 3.01
Group C—Control			
Week 1	4.97 ±0.56	4.43 ± 0.53	15.61 ± 2.54
Week 2	3.59 ± 0.22	3.71 ± 0.74	9.29 ± 1.54
Week 3	2.13 ± 0.38	2.41 ± 0.30	4.11 ± 0.88

**Table 2:** Mean inflammation score at two and three weeks.

	Early CS		Late CS		Control		*p*-Value
weeks	2 weeks	3 weeks	2 weeks	3 weeks	2 weeks	3 weeks	NS
Central	2.13 ± 0.47	1.00 ± 0.40	1.88 ± 0.47	1.25 ± 0.28	2.13 ± 0.47	0.63 ± 0.47	
Mid	2.00 ± 0.00	1.13 ± 0.25	1.88 ± 0.25	1.00 ± 0.40	2.00 ± 0.40	0.88 ± 0.25	
Lateral	1.75 ± 0.28	1.25 ± 0.28	2.00 ± 0.00	1.00 ± 0.57	2.00 ± 0.40	0.50 ± 0.40	
*p*-value	*p*-0.000		*p*-0.000		*p*-0.000		

## Data Availability

The data that support the findings of this study are available from the corresponding author [R.K.] upon reasonable request.
